# Valuable lessons-learned in transcriptomics experimentation

**DOI:** 10.1080/21541264.2015.1064195

**Published:** 2015-06-22

**Authors:** Oskar Bruning, Han Rauwerda, Rob J Dekker, Wim C de Leeuw, Paul F K Wackers, Wim A Ensink, Martijs J Jonker, Timo M Breit

**Affiliations:** 1 RNA Biology & Applied Bioinformatics research group; Swammerdam Institute for Life Sciences; Faculty of Science; University of Amsterdam; Amsterdam, the Netherlands; 2 MAD: Dutch Genomics Service & Support Provider; Swammerdam Institute for Life Sciences; Faculty of Science; University of Amsterdam; Amsterdam, the Netherlands

**Keywords:** Design for experimentation, experimental execution, experimental analysis, lessons-learned, Transcriptomics

## Abstract

We have collected several valuable lessons that will help improve transcriptomics experimentation. These lessons relate to experiment design, execution, and analysis. The cautions, but also the pointers, may help biologists avoid common pitfalls in transcriptomics experimentation and achieve better results with their transcriptome studies.

## Abbreviations

cDNA: copy DNA
DEG: differentially-expressed gene
FC: fold-change
NGS: next-generation sequencing
qPCR: quantitative polymerase chain reaction.

## Preamble

During the course of over a decade of transcriptomics experimentation, we have learned some valuable lessons. Now, at a turning point in transcriptomics experimentation, marked by the transition from microarray technology into next-generation sequencing, it seems timely to share these lessons with the life-sciences scientists that are increasingly including transcriptomics experiments in their research. Especially, since many issues raised here may have prevented transcriptomics to live up to its promises, particularly in the context of mechanistic studies.

We have organized the lessons- learned according to 3 leading topics: design, execution, and analysis of transcriptomics experimentation. As the distinction is not always clear, several lessons-learned have an effect on 2 or even all 3 elements of experimentation. The order is relatively arbitrary: all lessons are equally important, although some discussed elements have more profound effects on experiment interpretation than others.

We will restrict our lessons-learned to transcriptomics studies that aim to unravel subcellular molecular mechanisms. Biomarker studies have a different approach and objective and are not evaluated, although it is clear that many of the mentioned lessons likewise apply.

## Lessons-learned

### Design for Experimentation

Microarray technology and nowadays next-generation sequencing (NGS) have provided us with greatly improved “**detectors**” for investigating RNA levels in cell-based systems. Not only has it become possible to evaluate the gene-expression levels of many genes simultaneously by employing these transcriptomics techniques, but their results are also more quantitative than with the classical Northern blot and qPCR analysis (due to the variability of “housekeeping genes”). ^1^

One would expect that the introduction of such significantly improved detectors would have a major impact on how biologists **design** their new transcriptomics experiments. However, many biologists consider microarray technology merely a very high-throughput Northern blot and often still use “classical approaches” for their transcriptomics experiments. These are usually based on phenotypic endpoints, such as apoptosis or cell-cycle arrest, taken from common and accepted practice. Ignoring the impact of new detectors on experiment design and analysis may have serious consequences on the conclusions that one is allowed to draw based on such experiments.

The most obvious consequence is that if one investigates tens of thousands of genes, this will generate hundreds of thousands of observations, which will invariably lead to a considerable number of genes that are incorrectly implied to be involved in a process, due to chance combined with biological variability. Hence, **statistics** become extremely important in the analysis and thus in the design of the experiments. Tackling known and unknown confounding factors that are causing these false positives is the biggest challenge in transcriptomics. To this end, statistical countermeasures have to be implemented in all steps along the chain of experimentation. For instance, samples should be properly randomized, enough replicates should be included, appropriate statistical methods, such as false discovery rate correction, should be applied, and so on. In practice, optimal implementation of these statistical elements is often under duress due to budget constraints that limit numbers of replicates, absence of sufficient statistical expertise, or a desire to obtain publishable results, and so forth. Although many of these reasons often seem plausible and/or acceptable, the effect on the eventual outcome of the experiments is frequently underestimated. Moreover, since transcriptomics has increasingly become a hypothesis-generating approach, wrong conclusions may lead to flawed hypotheses, which in turn may lead to misdirected research. The obvious recommendation is that for proper design for transcriptomics experimentation, expert biostatisticians should be involved from the start to ensure a better chance of valid research. It is extremely important at this early design stage to foresee, as much as possible, the eventual required data analyses, as these regularly influence the experiment design.^2-4^

One key lesson, related to statistics, concerns **sample pooling**. For decades, biologists intuitively have pooled their samples. The reasoning is that “pooling averages out the differences between individual samples,” hence there will be less noise in the experiment. Although this is true,^5,6^ the differences between (replicated) samples are exactly what is needed for statistical power and inference. From a biological perspective, pooling of substantially different cells results in the generation of an artificial in-between cell type. Even though it has been somewhat of an uphill battle, currently more and more biologists are starting to realize that pooling in transcriptomics experimentation should be a conscious choice in the experimental design, because it can hamper biological interpretation.^5-8^

Another notion, related to the use of classical phenotypic endpoints, is that in order to achieve such a phenotypic endpoint, frequently quite **severe perturbations** are required.^9,10^ However, severe perturbations lead to severe transcriptome responses, which often represent a generic stress response rather than a specific reaction to the perturbation of interest.^10^ It seems that at a certain stress point, a cell will choose to activate its generic stress response. From that point on, the specific responses to a perturbation may no longer be present, not even hidden, behind the cloud of stress-induced noise.^10^ Ways to reduce these risks include, first of all, a precisely defined biological question and avoiding complicated set-ups with high-level and/or multiple biological questions and/or multiple experimental factors.

Furthermore, one should **tailor-make** each transcriptomics experiment to answer the specific biological question under study, instead of designing its setup based on classical phenotypic endpoints or common practice. This may include running small, technical and biological test experiments to determine the optimal experimental settings for a final experiment. Additionally, carefully planned range finding experiments are useful in determining the optimal location in the experiment design space.^11^ Designing range finding experiments forces a scientist to define its biological question or hypothesis quite narrowly; for example, instead of “which genes are involved in UV response?,” it would be something like “which genes in the nucleotide excision repair pathway respond to low-dose UV-C-induced DNA damage?.”

**Figure 1.**
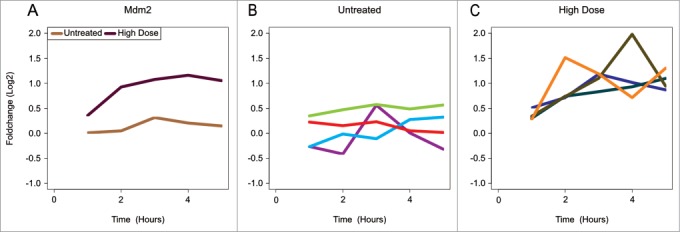
Smoothing effects of improper averaging over individuals. Time profiles of Log_2_ fold change compared to t = 0 of the *Mdm2* gene in skin derived from an *in vitro* mouse study. The mice were treated with UV-B at t = 0. (**A**) Averaged profiles over biological replicates for both treated and untreated samples. (**B**) Profiles of individual mice that were not treated. Different colors indicate individual mice. (**C**) Profiles of individual mice that were irradiated with a high dose of UV-B. Different colors indicate individual mice.

Unfortunately, range finding is not always sufficient. *In vivo* studies also need to be tested for **inter-individual differences**. If these are too diverse on a basal transcriptome level for the tissue under study, they cannot be considered replicates anymore. This effectively inhibits standard transcriptomics experimentation and a different experimental approach should be found. Examples of how improper replicates can be deceiving are our encounters, on several occasions, with very smooth looking profiles of average gene expression, suggesting a relation between the observed experimental factor and gene expression, whereas looking at the profiles from the underlying individual animals, no rhyme or reason could be detected (**Fig. 1**).

As a general suggestion, if one cannot afford high-quality ‘omics’ experiments, i.e., having a sufficiently **solid experiment design**, then such experiments should not be attempted. There are many transcriptomics experiments out there that have yielded sub-par results due to an incomplete experimental design, missing, for instance, essential controls due to budgetary reasons.^3,4^ In these cases, it would be better to set up the experiments on a smaller scale or using a different technique that has a solid design and lies within budgetary limits. Conversely, given the growing understanding of the role of miRNA in gene expression regulation in combination with good and affordable NGS technology for small RNA-seq, it seems good science to investigate both mRNA and miRNA transcriptomes in parallel within one experiment. We anticipate that this co-analysis will become common practice within the next few years.^12-14^ This is also true for including alternative splice variants in mRNA analysis, as they are increasingly recognized as an important biological principle. Given the fact that new long-read NGS techniques will allow for solid detection of splice variants, this also should become an integrated part of mRNA analysis.

### Experimentation Execution

One of the points that ties in with designing tailor-made experiments, is the fact that optimizing the **quality of starting material** can profoundly improve the experimental results. This includes: using single cells, homogenizing cell-populations, synchronizing cell-samples, and removing unwanted stressors. For instance, synchronization of the cultured cells at the start of the experiment and eliminating the commonly used excess of oxygen during *in vitro* experiments, lowers transcriptome variability and will result in more robust results.^10^ In general, it is often extremely difficult, if not impossible, to avoid confounding factors such as differential sample composition, e.g., due to infiltrating cells or time of day effects caused by circadian rhythm. Uncorrected confounding factors will limit the scope of an experiment or, in extreme cases, can render an experiment entirely useless.

Sensitive detectors are generally quite vulnerable to errors. This means that it is advisable to let experiments be executed by well-trained **transcriptomics experts**. The involved delicate laboratory equipment should be well-maintained and only be operated by these experts. The usually, relatively, relaxed laboratory attitude in molecular biology research can have negative effects on results obtained from such sensitive detectors. In the near future, it might be best if all transcriptomics experiments would be executed by recognized professionals organized in, certified, non-academic support facilities with a high level of application specialization.

Another technology-related notion concerns the continuing wish of biologists to include technical replicates in their transcriptomics experiments. This originates from an apparently indelible bad reputation from the early days of microarray technology, which is also reflected by the fact that many reviewers still demand validation of microarray-based results by qPCR. As a general rule for transcriptomics experimentation, biological variation heavily outweighs technological variation,^5^ so it is generally better to use biological replicates than technical ones.

**Probe-affinity** was long thought to be the major cause of differences in microarray signals between distinct probes investigating the same transcript. However, we now know that these so-called probe-affinity problems are related to sequence specific differences in cDNA synthesis and PCR amplification, enzymatic steps that are used in microarray technology. As these elements are also present in NGS, similar substantial differences in read coverage occur along transcripts.^15^ In other words, probe-affinity issues have been replaced by sequenceability, making any comparison between genes still unreliable.^16^

### Experiment Analysis

Obviously the outcome of any experiment analysis is highly dependent on proper design for experimentation combined with excellent experiment execution. As mentioned before, proper data analysis starts by anticipating the necessary data analyses during the experiment design phase. While everyone is familiar with the adagio “garbage in, garbage out,” many life-sciences researchers and bioinformaticians often still feel tempted to analyze poor data. Even though the reasons, such as expensive experimentation, or pressure to finish a PhD study in time, can be very persuasive, bioinformatics analysis of **poor data** invariably turns out to be an extremely time-consuming effort that rarely has a satisfying or scientifically sound outcome.^3,4^

Data analysis is still the domain of bioinformatics experts. Currently, we experience a trend in which simple-to-operate **software tools** allow biologists to analyze omics data themselves. Their increasing popularity among biologists is understandable from a perspective of both an apparent lack of skilled bioinformaticians and a desire to be independent. However, the fact that a scientist can operate a software tool does not mean that he or she can use it safely. Like driving a car, it helps if you have an expert instructor when you venture into treacherous traffic. Hence, bioinformatics-non-expert biologists can opt to parameterize optimistically and cherry pick their results. Consequently, they run the risk of using wrong parameters settings or analyses methods during their data analysis. Therefore, we recommend that at all times expert bioinformaticians at least help to set up the analysis workflow and preferably validate the workflow with synthetic data sets. Likewise, at the time of the result interpretation, expert genomics and bioinformatics guidance may help avoid wrong conclusions. This argument goes both ways, meaning that bioinformaticians should not interpret experiment results without the assistance of genomics and biology-domain experts. Much as we appreciate the desire for independence in each life-sciences researcher, we advocate a strict multidisciplinary approach when it comes to omics experimentation.^2,3,17^

One of the most prevalent hazards in data analysis is that biologists and/or bioinformaticians get lost in the transcriptomics data swamp. The sheer amount of data invariably leads to (apparent) remarkable observations. However, without a proper hypothesis these observations in **data-driven experiment analysis** are in essence just phenomena, irrespective of whether they are found by random data browsing or fancy data-correlating algorithms. Although a phenomenon can lead to an (interesting) hypothesis, more often it will lead to endless wading through the murky waters of transcriptomics data. Therefore, in the hypothesis-driven versus data-driven dilemma, we would advise to preferably use a hypothesis-driven approach in transcriptomics experimentation.^18^

Another consequence of sensitive genome-wide detectors is the fact that every response becomes visible. Choosing the relevant one is a challenge. This brings us to the burning question: Which transcriptome changes are **biologically relevant**? Despite the fact that we have statistical tools to determine whether a difference is statistically significant, we have no means to determine whether it is biologically relevant and, as such, all observed differences should be considered equally important. Yet, many scientists still use fold-change (FC) difference as a cut-off to select important differentially-expressed genes (DEGs), under the assumption that change equals importance. One could wonder whether this assumption is correct, given the fact that important regulating genes, like p53, are mostly expressed at a relatively low level and very often only show subtle differential expression.^9^ Therefore, one could equally persuasively argue that genes that are less important in gene expression regulation do not need to be rigorously controlled, given that control is expensive in terms of organization and energy. Advancing on that thought, it seems as if so-called “significant noise” exists in cellular organization. These are transcripts that are produced in a given situation and can be detected with statistical confidence, yet have no direct biological function just because, in some complex cases, it is more efficient to use a relaxed system for regulation than a strict one. To get a grip on functionality of mRNA transcripts, one could for instance check whether they are used for protein production by detecting the associated proteins.

With respect to interpretation of results, clustering of genes with differential expression has only limited use as it may only lead to the identification of genes that are controlled by the same mechanism, e.g., a common transcription factor. To increase the knowledge about pathways, which operate as cascades, gene set analysis is a better option for analysis, although this will not extend these pathways beyond the known gene sets. Another approach could be to consider RNA levels of all genes as a signature of a “**cell transcriptome state**.” Except for gene set analyses, most transcriptome analyses compare RNA levels of individual genes between different samples to identify DEGs. If one assumes that cells have a certain “state” that is defined by the relative presence of RNA between genes, one could compare the transcriptome state of samples rather than individual genes. Also, the constant presence and absence of gene expression is important and should be determined and incorporated in omics analyses. Of course, this implies modeling in a systems-biology approach, which currently seems to be out of reach when working with over 20,000 genes.

## Closing

It is evident that transcriptomics has brought us many new and exciting insights in the functioning of the cell, albeit often a message of confusing complexity. Moreover, we have no doubt that the approaching generation of omics technologies, which are on the brink of introduction, will bring us even more comprehensive knowledge and, undoubtedly, many new lessons. All together, we feel that the **paradigm shift** that has been preached from the dawn of the omics era has only just arrived.
